# Investigating the Impact of Perceived Discrimination on the Integration and Life Satisfaction of New Wave Turkish Immigrants Living in Germany

**DOI:** 10.1192/j.eurpsy.2023.380

**Published:** 2023-07-19

**Authors:** D. E. D. Cindik-Herbrüggen, E. Icer, G. Kara, S. Daryal

**Affiliations:** ^1^Psychiatry, Neuro-Psychiatrisches Zentrum Riem; ^2^N/A, Munich; ^3^belia seniorenresidenz wattenscheid, Bochum, Germany

## Abstract

**Introduction:**

Approximately 21.2 million people with im- migrant backgrounds live in Germany, which constituted 26% of its total population in 2020. Approximately 67% of immigrants are from European countries, including Turkey. Turks account for 13.2% of immigrants and constitute one of the largest immigrant groups (Statistisches Bundesamt, 2020). The integration processes and life satisfaction of new wave Turkish immigrants are differ from the first and second generation Turkish immigrants.

**Objectives:**

The aim of this study was to investigate the impact of perceived discrimination on their integration process and life satisfaction of new wave Turkish immigrants living in Germany.

**Methods:**

The Community Integration Measure (CIM), Satisfaction with Life Scale (SWLS), The Perceived Discrimination Scale (PDS) were used. Pearson correlation and Regression tests were used in our analyses to observe the differences in scale scores according to the variables. The relationship between the scale scores was analysed with the Pearson correlation test. The effect between the scale scores was analysed with the regression test.

**Results:**

The findings demonstrated that there was a significant negative relationship between perceived discrimination and social integration (p=0,05) as well as life satisfaction (p=0,05). In addition, there was a significant and positive relationship between social integration and life satisfaction of new wave Turkish immigrant participants.

**Conclusions:**

The integration process and life satisfaction of new wave Turkish immigrants decreased when they perceive discrimination in their daily lives. However, the level of life satisfaction increased when they integrate into German society without any perceived discrimination.

**Disclosure of Interest:**

None Declared

